# Radiosensitization of colorectal carcinoma cell lines by histone deacetylase inhibition

**DOI:** 10.1186/1748-717X-1-25

**Published:** 2006-08-03

**Authors:** Kjersti Flatmark, Ragnhild V Nome, Sigurd Folkvord, Åse Bratland, Heidi Rasmussen, Mali Strand Ellefsen, Øystein Fodstad, Anne Hansen Ree

**Affiliations:** 1 Department of Tumor Biology, Rikshospitalet-Radiumhospitalet Medical Center, University of Oslo, 0310 Oslo, Norway; 2 Department of Surgical Oncology, Rikshospitalet-Radiumhospitalet Medical Center, 0310 Oslo, Norway; 3 Department of Medical Oncology and Radiotherapy, Rikshospitalet-Radiumhospitalet Medical Center, 0310 Oslo, Norway; 4 Department of Radiation Biology, Rikshospitalet-Radiumhospitalet Medical Center, 0310 Oslo, Norway

## Abstract

**Background:**

The tumor response to preoperative radiotherapy of locally advanced rectal cancer varies greatly, warranting the use of experimental models to assay the efficacy of molecular targeting agents in rectal cancer radiosensitization. Histone deacetylase (HDAC) inhibitors, agents that cause hyperacetylation of histone proteins and thereby remodeling of chromatin structure, may override cell cycle checkpoint responses to DNA damage and amplify radiation-induced tumor cell death.

**Methods:**

Human colorectal carcinoma cell lines were exposed to ionizing radiation and HDAC inhibitors, and cell cycle profiles and regulatory factors, as well as clonogenicity, were analyzed.

**Results:**

In addition to G_2_/M phase arrest following irradiation, the cell lines displayed cell cycle responses typical for either intact or defective p53 function (the presence or absence, respectively, of radiation-induced expression of the cell cycle inhibitor p21 and subsequent accumulation of G_1 _phase cells). In contrast, histone acetylation was associated with complete depletion of the G_1 _population of cells with functional p53 but accumulation of both G_1 _and G_2_/M populations of cells with defective p53. The cellular phenotypes upon HDAC inhibition were consistent with the observed repression of Polo-like kinase-1, a regulatory G_2_/M phase kinase. Following pre-treatment with HDAC inhibitors currently undergoing clinical investigation, the inhibitory effect of ionizing radiation on clonogenicity was significantly amplified.

**Conclusion:**

In these experimental models, HDAC inhibition sensitized the tumor cells to ionizing radiation, which is in accordance with the concept of increased probability of tumor cell death when chromatin structure is modified.

## Background

Standard treatment of rectal cancer that by clinical or radiological assessment reveals locally advanced growth within the pelvis involves preoperative radiotherapy aimed at down-staging the tumor, to facilitate subsequent surgical excision [1,2]. However, tumor response to preoperative therapy varies greatly from pathological complete response to lack of objective response, warranting the use of experimental models to assay the efficacy of molecular targeting agents in rectal cancer radiosensitization.

The combination of radiotherapy and chemotherapy is advocated primarily because of the independent effect of each modality. Chemotherapeutics enhance radiocytotoxicity by means of increasing the initial DNA damage, inhibiting DNA repair, or slowing down cellular repopulation during fractionated radiotherapy, which are mechanisms that essentially depend on cell cycle synchronization of the tumor cell population [3]. Theoretically, such synchronization is achieved when sub-lethal DNA damage is applied to the tumor cells, by means of activation of signaling pathways that are rapidly manifested as arrests at cell cycle checkpoints [4].

Massive insult on DNA, such as double-strand DNA breaks following cellular exposure to ionizing radiation, may induce checkpoint responses in essentially any phase of the cell cycle [4], ultimately leading to the outcome of cell survival if DNA is properly repaired or, if not, cell death [5]. The signaling pathway via the tumor-suppressor protein p53, the primary regulator of the G_1 _checkpoint, is often defective in human solid tumors. In tumor cells with intact p53 function, however, DNA damage leads to rapid p53 stabilization and subsequent induction of the G_1 _phase inhibitor p21 [5]. The mechanism of DNA damage-activated G_2 _checkpoint signaling, initiated by ATM, involves inhibition of the enzymatic activity of Polo-like kinase-1 (Plk1) and subsequent delay in activation of the G_2_/M transition kinase [6]. We have previously found that cell cycle arrest of breast carcinoma cell lines at the G_2_/M boundary comprises repression of the gene for Plk1, *PLK *[7-9].

A variety of pharmacological compounds, designed to target cell cycle regulatory mechanisms, have been shown to override the DNA damage defense response that prevents mitotic entry [10]. Such agents may have therapeutic potential as radiosensitizers by facilitating cell death by mitotic catastrophe, and a wide array of compounds are undergoing clinical development [11].

Drugs that modify the cellular chromatin structure may also radiosensitize tumor cells. Taxanes, which disrupt chromatin structure and chromosome segregation in mitotis, are currently utilized clinically as radiosensitizers in treatment of non-small cell lung cancer and head-and-neck cancer [12]. Cellular treatment with HDAC inhibitors causes hyperacetylation of histone proteins, which leads to remodeling of chromatin structure [13]. In addition to this, the pertubation by HDAC inhibitors of cell cycle checkpoint signaling [14] might constitute the cellular mechanism by which these compounds enhance tumor cell sensitivity to radiation treatment. Currently, seven HDAC inhibitors are under investigation in clinical trials [15].

In a previous report we compared cell cycle responses of a human breast carcinoma cell line to ionizing radiation and HDAC inhibition [7]. The cell line we used required rather high concentrations of the HDAC inhibitor, trichostatin A (TSA), to reveal histone acetylation. Moreover, we chose to treat the cell line with a high radiation dose (8 Gy) to possibly achieve clearly defined effects on the cell cycle phenotype. In these breast carcinoma cells, the G_2 _phase responses to ionizing radiation were closely similar to those observed upon TSA treatment [7].

The frequency of *TP53 *mutations in colorectal cancer is 40–50% [16]. Hence, in the present study we have compared colorectal carcinoma cell lines with wild-type or mutated *TP53*, to evaluate the use of HDAC inhibitors in combination with ionizing radiation in rectal cancer. As valid experimental conditions for rectal cancer therapy, we measured inhibitory effects of ionizing radiation on clonogenicity after exposure to radiation doses of 2 or 5 Gy, which are fractionation doses used in preoperative treatment of locally advanced disease [1,17], and the possible radiosensitization by suberoylanilide hydroxamic acid (SAHA; currently licensed as vorinostat) or the benzamide MS-275, which are HDAC inhibitors in clinical development [15].

## Methods

### Cell lines and experimental treatments

The origin of the human colorectal carcinoma cell lines is delineated previously [18]. The HCT116 and SW620 cell lines were purchased from ATCC (Manassas, VA, USA). The Co115 cell line was obtained from Dr. B. Sordat (Swiss Institute of Experimental Cancer Research, Epalinges, Switzerland), whereas the KM20L2 cell line was provided by Dr. M. R. Boyd (National Cancer Institute, Frederick, MD, USA). All cell lines were cultured in RPMI 1640 medium supplemented with 10% fetal bovine serum and 2.0 mM glutamine. High-energy radiation from a ^60^Co source was delivered at a rate of approximately 0.6 Gy/minute. The unirradiated control cells were simultaneously placed in room temperature to obtain comparable conditions. The commercially available HDAC inhibitors TSA and SAHA were obtained from Sigma-Aldrich Norway (Oslo, Norway), whereas the HDAC inhibitor MS-275 was a generous gift from Schering AG (Berlin, Germany).

### Flow cytometry analysis

Cells were harvested in ice-cold phosphate-buffered saline, centrifuged, and fixed in 100% methanol. To determine the fractions of cells in G_1_, S, and G_2_/M phases from the cell cycle distribution, the cells were stained with 1.5 μg/ml Hoechst 33258 in phosphate-buffered saline and analyzed in a FACStar+ flow cytometer (Becton Dickinson, San Jose, CA, USA), as described previously [8].

### Western blot analysis

Protein expression was measured by means of standard Western blot technique, as described previously [8], and all experiments were performed two or three independent times. The membranes were immunostained with designated primary antibodies obtained from Zymed Laboratories Inc. (San Francisco, CA, USA), Santa Cruz Biotechnology (Santa Cruz, CA, USA), Calbiochem/Merck Biosciences Ltd. (Nottingham, UK), or Upstate (Lake Placid, NY, USA). These were anti-Plk1 (Zymed; 33–1700), anti-p53 (SC-6243), anti-Cyclin D1 (SC-20044), anti-p21 (SC-6246), anti-α-tubulin (Calbiochem; CP06), anti-acetyl-histone H3 (Upstate; 06–599), and anti-acetyl-histone H4 (Upstate; 06–866), respectively.

### Northern blot analysis

Expression of RNA was measured by means of standard Northern blot technique, as described previously [8]. The human cDNA clone for *PLK *was obtained from RZPD Deutsches Ressourcenzentrum für Genomforschung GmbH (Berlin, Germany). The human cDNA probe for *CCND1 *was a gift from Dr. D. Beach (Howard Hughes Medical Institute, Cold Spring Harbor, NY, USA), and the human cDNA probe for *CDKN1A *was a gift from Dr. B. Vogelstein (The John Hopkins University School of Medicine, Baltimore, MD, USA). To evaluate the amounts of RNA loaded, the filters were rehybridized to a kinase-labeled oligonucleotide probe complementary to nucleotides 287–305 of human 18S rRNA.

### Assessment of clonogenicity

Clonogenic regrowth efficiency was determined by plating single cells suspended in medium. The cells were left for 6 hours to allow attachment to the plastic before the medium was replaced by media with or without HDAC inhibitors. Following 18 hours incubation, the media were changed to fresh medium (without any drug) and the cells immediately irradiated. The appropriate plating density was aimed to produce 20–40 surviving colonies in each well of six-well culture plates. After incubation for 7 days, the cell colonies were fixed and stained with 0.1% crystal violet. Colonies of ≥ 50 cells were counted for computing of surviving fraction. At least three parallel samples were scored in three to five repetitions performed for each treatment condition.

## Results

### Cell cycle responses to ionizing radiation

**Figure 1:**
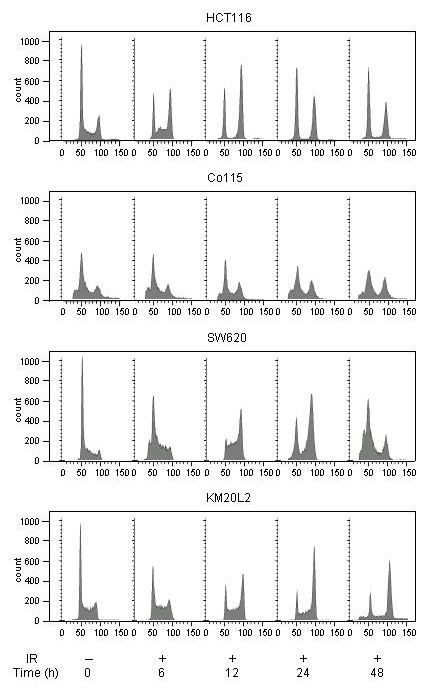
**Cell cycle profiles following exposure to ionizing radiation (IR)**. Four colorectal carcinoma cell lines (HCT116, Co115, SW620, and KM20L2) were exposed to 8 Gy of IR (+) and further incubated for the indicated time periods before cellular DNA contents were determined by flowcytometry analysis gated for Hoechst 33258 fluorescence. Cells with DNA contents characteristic for G_1 _and G_2_/M phase cells were found in channel numbers ~50 and 90–100 along the x axes, respectively. Scales indicating cell counts (y axes) are provided.

Four colorectal carcinoma cell lines (HCT116, Co115, SW620, and KM20L2) were initially observed for 48 hours for cell cycle responses to ionizing radiation (8 Gy). As seen from Figure 1, the HCT116 and SW620 cell lines displayed typical patterns of cell cycle redistribution for cells with intact (HCT116) or defective (SW620) p53 function, respectively. Irradiated HCT116 cells were arrested in G_1 _phase shortly after DNA damage, while S phase cells were progressing into G_2_/M phase (observe the S phase shift at 6–12 hours). A distinct accumulation of G_2_/M phase cells was seen during the remaining observation period (Figure 1, upper panel). In contrast, radiation exposure of the SW620 cell line resulted in depletion of G_1 _phase cells but instead G_2_/M phase delay, which apparently persisted for a period 24 hours or longer after DNA damage but did not seem to be plenary, as a new G_1 _population was observed after 24 hours (Figure 1, third panel from top).

**Figure 2:**
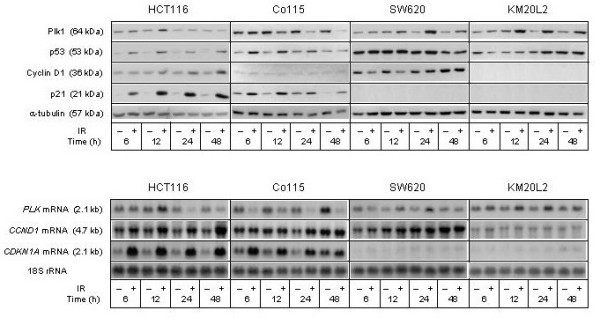
**Cell cycle regulatory factors following exposure to ionizing radiation (IR)**. Four colorectal carcinoma cell lines (HCT116, Co115, SW620, and KM20L2) were exposed (+) to 8 Gy of IR, or left unexposed (-), and further incubated for the indicated time periods before analysis. Upper panel: Protein expression levels of Plk1, p53, Cyclin D1, and p21 were analyzed by Western blot immunostaining, using α-tubulin as protein loading control. Lower panel: mRNA expression levels of *PLK*, *CCND1*, and *CDKN1A *were analyzed by Northern blot hybridization, using 18S rRNA as RNA loading control.

The responses of regulatory proteins of the G_1 _and G_2_/M cell cycle phases to ionizing radiation were also followed (Figure 2) to observe whether these might correlate to the changes in cell cycle redistribution. In irradiated HCT116 cells, rapid induction of the G_1 _phase inhibitor p21 and its mRNA (*CDKN1A*) was observed, consistent with the immediate stabilization of p53 following DNA damage. Interestingly, expression of the principal G_1 _phase cyclin, Cyclin D1, seemed to be up-regulated by ionizing radiation as well, but with much lower amplitude and slower kinetics than p21. The SW620 cells showed complete absence of G_1 _checkpoint-activated characteristics (p53 and p21 responses), and Cyclin D1 was rather down-regulated, though transiently. In contrast to what we have previously observed in various breast carcinoma cell lines, in which expression of the G_2_/M phase kinase Plk1 has been found to be transiently down-regulated following radiation exposure [7-9,19], Plk1 expression was found to be increased above control level in irradiated SW620 cells and possibly also in the HCT116 counterparts (Figure 2).

Following radiation exposure of the wild-type *TP53 *Co115 cell line, the percentage of G_2 _phase cells was gradually increasing, while a distinct G_1 _population was maintained during the observation period (Figure 1, second panel from top). In these cells, p53 stabilization and resulting induction in *CDKN1A *mRNA and p21 protein as well as repression of *PLK *mRNA and Plk1 protein were seen (Figure 2). These response profiles to DNA damage were highly correlated to the observed changes in cell cycle redistribution. The KM20L2 cell line displayed lack of G_1 _checkpoint protein responses but increased Plk1 expression upon irradiation (Figure 2). As seen from the lower panel of Figure 1, this cell line showed distinct DNA damage-induced G_2_/M phase arrest but with a small G_1 _population present during the entire observation period.

### Cell cycle responses to TSA

**Figure 3:**
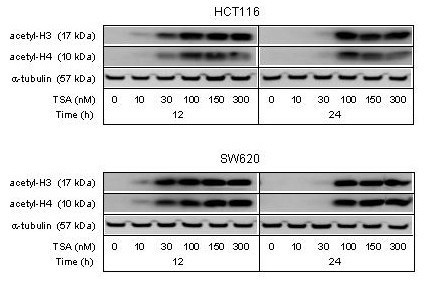
**Histone acetylation by TSA**. The HCT116 and SW620 cell lines were treated with TSA in increasing concentrations, and protein extracts prepared after 12 and 24 hours of incubation were analyzed by Western blot immunostaining with antibodies against acetylated histones H3 (acetyl-H3) and H4 (acetyl-H4). α-tubulin was measured as protein loading control.

Since cell cycle responses associated with intact or defective p53 function were typically displayed by the HCT116 and SW620 cell lines, respectively, effects of HDAC inhibition by TSA were analyzed in these particular cell lines. Tumor cell sensitivity to HDAC inhibitors may vary along a wide concentration range and should be considered highly cell line-specific. Thus, effects of increasing concentrations of TSA (10–300 nM) on histone acetylation status of the HCT116 and SW620 cell lines were determined. As seen from Figure 3, levels of acetylated core histones H3 and H4 were substantially induced after 12 and 24 hours incubation with TSA concentrations above 30 nM, suggesting that TSA in concentrations of 30–100 nM for a treatment period of 12–24 hours might be appropriate for further mechanistic studies.

**Figure 4:**
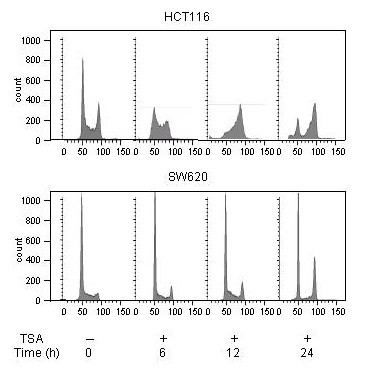
**Cell cycle profiles upon TSA treatment**. The HCT116 and SW620 cell lines were treated (+) with 100 nM TSA and further incubated for the indicated time periods before cellular DNA contents were determined by flow cytometry analysis gated for Hoechst 33258 fluorescence. Cells with DNA contents characteristic for G_1 _and G_2_/M phase cells were found in channel numbers ~50 and 90–100 along the x axes, respectively. Scales indicating cell counts (y axes) are provided.

Pharmacological inhibition of HDAC activity has been shown to cause cell cycle arrest in the G_2_/M phase in a variety of tumor cell lines [7,20-23], resembling DNA damage-induced G_2 _checkpoint response to ionizing radiation. Interestingly, TSA treatment (100 nM) of the HCT116 and SW620 cell lines for a period of 0–24 hours resulted in cell cycle responses highly different from the irradiated phenotypes. In the HCT116 cells, complete depletion of G_1 _phase cells followed by arrest of cells in G_2_/M phase was observed, before a new G_1 _population appeared after 24 hours of TSA incubation (Figure 4, upper panel). Moreover, TSA-treated SW620 cells were instantly arrested in G_1 _phase, while S phase cells were gradually progressing into G_2_/M phase. A distinct accumulation of G_2_/M phase cells was seen during the entire observation period (Figure 4, lower panel). Hence, in both HCT116 and SW620 cells, TSA treatment was associated with redistribution of cell populations into radiosensitive cell cycle phases (G_1 _or G_2_/M).

**Figure 5:**
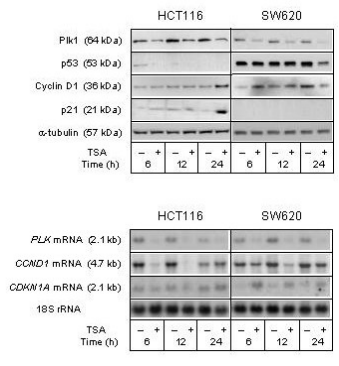
**Cell cycle regulatory factors upon TSA treatment**. The HCT116 and SW620 cell lines were treated (+) with 100 nM TSA, or left untreated (-), and further incubated for the indicated time periods before analysis. Upper panel: Protein expression levels of Plk1, p53, Cyclin D1, and p21 were analyzed by Western blot immunostaining, using α-tubulin as protein loading control. Lower panel: mRNA expression levels of *PLK*, *CCND1*, and *CDKN1A *were analyzed by Northern blot hybridization, using 18S rRNA as RNA loading control.

Consistent with the G_2_/M phase accumulation of both cells lines, TSA-dependent Plk1 repression was seen (Figure 5), similar to what we have observed previously in a breast carcinoma cell line [7]. From below detection, p21 expression seemed to be induced 24 hours after addition of TSA to the HCT116 cells. In contrast, p53 expression appeared to be repressed in the SW620 cells 24 hours after TSA addition (Figure 5). These TSA-dependent characteristics have previously been found to coincide in breast carcinoma cells [7]. Apart from *PLK *mRNA, apparent TSA-associated changes in mRNA levels did not convincingly translate into the respective cell cycle proteins (Figure 5).

### Ionizing radiation and HDAC inhibition by TSA – clonogenicity

**Figure 6:**
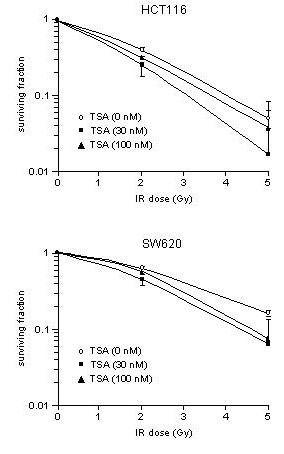
**TSA modulates clonogenic regrowth upon cellular exposure to ionizing radiation (IR)**. The HCT116 and SW620 cell lines were exposed to increasing IR doses without (○) or following pre-treatment for 18 hours with TSA in concentrations of 30 nM (■) or 100 nM (▲), to determine relative colony formation compared to the unirradiated situation (mean ± SEM, n = 3).

Next, the HCT116 and SW620 cell lines were exposed to therapeutically utilized doses of ionizing radiation (2 and 5 Gy) to determine clonogenic survival. Cell cycle responses to 5 Gy of ionizing radiation, assessed as time-dependent redistribution of cell cycle phases and expression of corresponding regulatory proteins (data not shown), were essentially indistinguishable from those to 8 Gy described above. As shown by Figure 6, the HCT116 cells showed surviving fractions of ~0.4 and 0.07–0.1 with 2 and 5 Gy, respectively, whereas relative SW620 colony formation upon exposure to those doses were ~0.6 and ~0.15, respectively.

Moreover, the possible radiosensitizing effect of TSA, essentially by amplifying the inhibitory effect of ionizing radiation on clonogenicity, was measured. Based on the histone acetylation data (Figure 3) and the observed redistribution of cell cycle phases (Figure 4), we chose to analyze the cell lines upon incubation with 30 and 100 nM concentrations of TSA for 18 hours before the HDAC inhibitor was removed and the cells irradiated. With these incubation conditions, unirradiated HCT116 cells showed surviving fractions of ~0.5 and ~0.35 with 30 and 100 nM TSA, respectively, whereas relative SW620 colony formation was ~0.6 with both TSA concentrations. As seen from Figure 6, the cytotoxic effect of ionizing radiation on both HCT116 and SW620 cell lines seemed to be amplified by TSA, but interestingly more pronounced with the lower concentration.

### Ionizing radiation and HDAC inhibition by SAHA or MS-275 – clonogenicity

**Figure 7:**
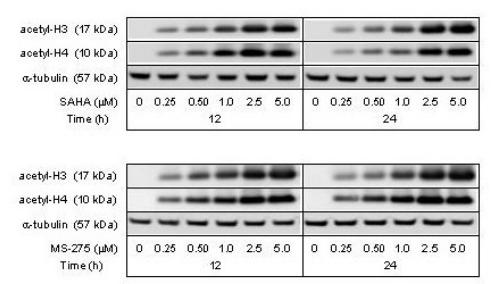
**Histone acetylation by SAHA and MS-275**. The HCT116 cells were treated with SAHA (upper panel) or MS-275 (lower panel) in increasing concentrations, and protein extracts prepared after 12 and 24 hours of incubation were analyzed by Western blot immunostaining with antibodies against acetylated histones H3 (acetyl-H3) and H4 (acetyl-H4). α-tubulin was measured as protein loading control.

Finally, the HCT116 cells were also treated with two HDAC inhibitors that are currently in clinical investigation (SAHA and MS-275) to determine if those might cause radiosensitization. As shown by Figure 7, levels of acetylated histones H3 and H4 were induced in a concentration-dependent manner after 12 and 24 hours exposure to SAHA or MS-275 (both 0.25–5.0 μM).

**Figure 8:**
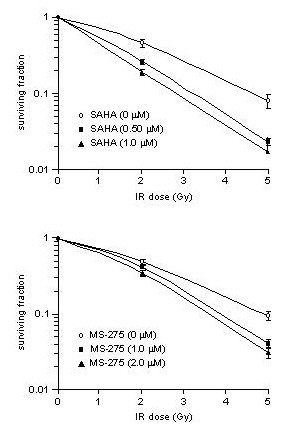
**SAHA and MS-275 modulate clonogenic regrowth upon cellular exposure to ionizing radiation (IR)**. The HCT116 cells were exposed to increasing IR doses without (○) or following pre-treatment for 18 hours with SAHA in concentrations of 0.50 μM (■) or 1.0 (M (▲) (upper panel) or MS-275 in concentrations of 1.0 μM (■) or 2.0 μM (▲) (lower panel), to determine relative colony formation compared to the unirradiated situation (mean ± SEM, n = 3–5).

Theoretically, chemotherapeutics enhance radiocytotoxicity within concentration ranges that apply sub-lethal DNA damage to the tumor cells. Upon incubation of the HCT116 cells for 18 hours, 10–25% inhibition of colony formation was achieved with SAHA and MS-275 within low micromolar concentration ranges (0.50–1.0 μM and 1.0–2.0 μM, respectively). And as seen from Figure 8, clonogenicity of irradiated HCT116 cells was significantly reduced by both compounds under these incubation conditions.

## Discussion

In this report we have compared cell cycle response profiles of human colorectal carcinoma cell lines to ionizing radiation and HDAC inhibition. In addition to G_2_/M phase arrest following radiation exposure, the cell lines displayed cell cycle responses typical for either intact or defective p53 function. In contrast to the profiles induced by irradiation, HDAC inhibition was associated with complete depletion of the G_1 _phase population of cells with functional p53 but accumulation of both G_1 _and G_2_/M phase populations of cells with defective p53. Moreover, histone acetylation was followed by significant reduction in clonogenic regrowth of irradiated cells, irrespective of p53 status. This observation is in accordance with the concept of increased probability of tumor cell death when the chromatin structure is modified.

Each cell line's p53 status was also confirmed by sequence analysis of the *TP53 *gene, by means of methodology described previously [7]. In both cell lines with *TP53 *mutation (SW620 and KM20L2), a base substitution of A for a G nucleotide in codon 273, resulting in change of amino acid Arg to His, was detected (data not shown). According to the International Agency for Research on Cancer's *TP53 *Mutation Database [24], this particular base substitution represents ~5% of all *TP53 *mutations recorded in colorectal carcinomas. The frequency of mutations in the hotspot codon 273 in an international cohort of colorectal carcinoma patients was recently reported to be 8% [25], which may be regarded as a substantial fraction of patients with *TP53*-mutated colorectal tumors.

In a variety of tumor cell models, pharmacological inhibition of HDAC activity has been shown to cause redistribution of cell cycle profiles resembling G_2 _checkpoint responses to DNA damage [7,20-23]. Although accumulation in G_1 _phase has been reported [22,23,26,27], induction of the G_1 _phase inhibitor p21 and concomitant hypophosphorylation of the retinoblastoma protein upon HDAC inhibition have been shown to occur without subsequent G_1 _checkpoint arrest [20]. Our findings do not clarify the issue of whether p21 may be involved. In the SW620 cells, TSA treatment was associated with maintained G_1 _population in the absence of any p21 expression. Furthermore, the finding that a G_1 _population reappeared in TSA-treated HCT116 cells is more likely due to release of cells arrested in G_2_/M phase than to a concurrent p21 induction.

Although p21 as well as the principal G_1 _phase cyclin, Cyclin D1, are considered targets for regulation by HDAC inhibition [28-30], regulatory responses of these cell cycle factors to TSA were not convincingly displayed by HCT116 or SW620 cells. In contrast, repression of the G_2_/M phase kinase Plk1 was clearly observed in both TSA-treated cell lines, consistent with the G_2_/M phase accumulation concurrently seen. The TSA-directed decline in *PLK *mRNA expression is in accordance with our previous finding [7]. *PLK *is among several genes, encoding mitotic regulators, of which mRNA expression is down-regulated following activation of the G_2 _checkpoint [31]. Apart from the Co115 cell line and contrary to our observations in various breast carcinoma cells lines [7-9,19], however, Plk1 was found to be up-regulated rather than down-regulated upon irradiation.

In tumor cell lines, cytotoxicity of chemotherapeutics and the anti-Her2 antibody trastuzumab has been found increased by the presence of SAHA and MS-275 [21,32,33]. Recently, MS-275 was also shown to sensitize tumor cell lines to the growth-inhibitory effect of retinoic acid [34]. Among HDAC inhibitors in clinical investigation, five have been reported to act as radiosensitizers in preclinical models [26,35-40]. Interestingly, in animal models, topical skin application of HDAC inhibitors significantly suppressed cutaneous side effects of radiotherapy [41], suggesting that the contemporary approach of molecularly targeted therapy may be utilized to increase the therapeutic ratio between the tumor and surrounding normal tissues in radiotherapy. To our knowledge, the present report is the first to study HDAC inhibition as radiosensitizing strategy with therapeutically relevant radiation doses in colorectal cancer.

In contrast to what was observed with SAHA and MS-275, a threshold concentration of TSA (30–100 nM) seemed to be necessary to obtain cellular acetylation of core histones H4 and H3. Histone acetylation was clearly present with 30 nM TSA after 12 hours incubation but absent after 24 hours, whereas with 100 nM, hyperacetylation was maintained after 24 hours. Identical observations were done in other colorectal carcinoma cell lines (data not shown). Yet, following pre-treatment for 18 hours, the lower TSA concentration (30 nM) was found to sensitize both cell lines (HCT116 and SW620) to the inhibitory effect of ionizing radiation on clonogenicity, while the higher concentration (100 nM) seemed less efficacious. A similar phenomenon has been reported after experimental *in vivo *use of MS-275, as inhibition of osteolytic bone metastases seemed to be more efficient with the lower therapy dose [42]. It has previously been shown that TSA also acts via mechanisms involving acetylation of non-histone proteins, which might be of consequence for TSA-induced cytotoxicity [22]. Moreover, it has been suggested that different classes of HDAC inhibitors may cause differential protein acetylation and, to a certain degree, differential gene expression [43,44]. Such differences in effector mechanisms might account for the apparent feature of TSA *contra *SAHA and MS-275 to whether the histone acetylation status might directly predict the compounds' efficacy of sensitizing the tumor cells to DNA-damaging therapy.

While TSA has shown excessive toxicity under *in vivo *conditions, both SAHA and MS-275 have reached clinical investigation [45-47]. The development and early therapeutic utilization of such compounds demand biomarker(s) that may provide direct insight into their mode of action. The complexity of effector mechanisms involved with TSA is probably a main reason why this agent is not feasible to monitor and, hence, use safely in the *in vivo *setting.

## Conclusion

There is strong scientific evidence that chromatin-remodeling drugs may radiosensitize tumor cells. The present report indicates that histone acetylation is associated with enhanced radiocytotoxicity in colorectal carcinoma cell lines, irrespective of their *TP53 *mutation status. Whether such information might translate into strategies to improve radiotherapy outcome in rectal cancer, requires further experimental approaches but hints, if anything, at an appealing concept.

## Competing interests

The author(s) declare that they have no competing interests.

## Authors' contributions

KF participated in the design of the study, and contributed to data acquisition, data analysis and drafting the manuscript. RVN participated in performing the Northern and Western blot analyses. SF performed the clonogenicity analyses. ÅB carried out the Northern blot analyses. HR carried out the Western blot analyses. MSE carried out the flow cytometry analyses. ØF participated in the study design and helped to draft the manuscript. AHR participated in the design of the study, contributed to data acquisition and analysis, and drafted the manuscript. All authors read and approved the final manuscript.
